# Plasma Metabolomic and Intestinal Microbial Analyses of Patients With Severe Aplastic Anemia

**DOI:** 10.3389/fcell.2021.669887

**Published:** 2021-08-23

**Authors:** Yuanyuan Shao, Weiwei Qi, Xiaomei Zhang, Ningyuan Ran, Chunyan Liu, Rong Fu, Zonghong Shao

**Affiliations:** Department of Hematology, General Hospital of Tianjin Medical University, Tianjin, China

**Keywords:** aplastic anemia, metabolomics, microbiota, plasma, gut

## Abstract

Aplastic anemia results from bone marrow failure caused by an autoimmune abnormality, but the pathogenesis of severe aplastic anemia (SAA) is not well characterized. To identify potential metabolic markers of SAA and to further elucidate the pathogenetic mechanisms of SAA, we performed a metabolomic study of plasma samples and characterized the intestinal microbiota of patients with SAA and healthy controls. Patients with SAA had more Enterobacteriales and Lactobacillales, but fewer Bacteroidales, Clostridiales, and Erysipelotrichales than healthy controls. At the species level, the abundances of *Escherichia coli* and others including *Clostridium citroniae* were higher, whereas those of *Prevotella copri*, *Roseburia faecis*, and *Ruminococcus bromii* were lower. Eight metabolites showed significantly different plasma concentrations in the SAA and healthy control groups. Coumaric acid, L-phenylalanine, and sulfate were present at higher concentrations in the SAA group; whereas L-glutamic γ-semialdehyde, theobromine, 3a, 7a-dihydroxy-5b-cholestane, γ-δ-dioxovaleric acid, and (12Z)-9, 10-dihydroxyoctadec-12-enoic acid were present at lower concentrations. In conclusion, patients with SAA show abnormalities in both their plasma metabolomes and intestinal microbial compositions. These differences might reflect the molecular mechanisms involved in the defective immunity that characterizes SAA.

## Introduction

Severe aplastic anemia (SAA) is a class of hematological diseases that is characterized by pancytopenia and bone marrow failure. Immunosuppressive therapy (IST) using antithymocyte globulin and cyclosporine A has been used as the first-line treatment for patients with SAA, and this improves their prognosis (Killick et al., 2016). SAAs are currently considered to be immune disorders of the bone marrow hematopoietic cells that involve damage resulting from hyperfunction of cytotoxic T lymphocytes (Fogarty et al., 2003; Maciejewski and Risitano, 2003). Patients with SAA also show an imbalance of T-helper (Th)1 and Th2 cells (Solomou et al., 2006), fewer regulatory T cells (Tregs) (Solomou et al., 2007), abnormally activated myeloid dendritic cells (mDCs) (Nakao, 2013; Ogawa, 2016), and abnormally high concentrations of Th1-type cytokines (Gidvani et al., 2007). However, the precise immunopathogenesis of SAA is unclear.

In recent studies, abnormal metabolism and abnormal composition of the intestinal microbiota have been shown to play important roles in autoimmune diseases. Abnormal composition of the gut microbiota is linked to a number of human diseases (Qin et al., 2012; Dodd et al., 2017). Furthermore, metabolomics studies have shown differences in concentrations of key metabolites in hematological diseases (Chen et al., 2014). The immune-mediated defects in bone marrow hematopoiesis have been shown to be triggered by certain types of chronic inflammation or infection (Maciejewski et al., 2000; Chihara et al., 2018). Furthermore, alterations to the intestinal microbiota and chronic enteritis may provide persistent stimuli that induce and sustain the immune pathophysiology (Espinoza et al., 2016). A previous case report that described a 30-years-old male patient with refractory SAA revealed an inadvertently good hematological response to the treatment of intestinal inflammation, which supports a hypothetical but plausible pathogenic association (Zhao et al., 2020). However, although evidence for associations between the intestinal microbiota, diseases, and symptoms is accumulating, the design of novel therapies that are based on these links necessitates much fuller knowledge of the roles of these intestinal microorganisms.

In this study, we performed a metabolomic study of the plasma and characterized the intestinal microbiota of patients with SAA and healthy controls to identify potential metabolic markers and further elucidate the pathogenetic mechanisms of SAA.

## Materials and Methods

### Study Participants

A total of 10 patients with SAA that was diagnosed at the Hematology Department of Tianjin Medical University were enrolled between January 2018 and January 2019 (six men and four women; median age = 56.5 years, range = 17–77 years). In addition, 14 healthy adults (3 men and 11 women; median age = 43.5 years, range = 26–63 years) were recruited as the control group.

A diagnosis of SAA was made according to the criteria of the International AA Study Group (Marsh et al., 2009). Bone marrow biopsy and aspiration for morphology and cytogenetics were performed before enrolment. All the patients were tested for paroxysmal nocturnal hemoglobinuria (PNH) using a flow cytometric assay, but no PNH clones were identified. None of the participants had taken antibiotics or probiotics within the 3 months prior to admission. The clinical data for all of the participants are shown in Table 1. There were no statistically significant differences in the clinical data between the two groups (*p* > 0.05), and the severity of the disease was similar in all the patients. The study was approved by the Ethics Committee of the Tianjin Medical University.

**TABLE 1:** Clinical data for all the participants.

	SAA group	Control group
N	10	14
Females (%)	40.0	78.6
Median age	56.5	43.5
Age range	17–77	26–63
Hemoglobin (g/L)	68.30 ± 2.32	130.43 ± 1.68
Platelet (× 10^9^/L)	12.60 ± 1.44	266.86 ± 14.05
Neutrophil (× 10^9^/L)	0.61 ± 0.09	3.27 ± 0.24
Reticulocyte (× 10^9^/L)	13.80 ± 0.95	55.71 ± 4.60

### Intestinal Microbial Analysis

#### Sample Collection and Preservation

None of the participants had undergone antibiotic or IST within the 3 months preceding admission. Stool samples were collected within 2 h after the participants were consuming their standard diet. Samples from the middle and rear of the stool were collected using sterile cotton swabs. Half a milliliter of each sample of feces was collected into a 1.5-mL sterile Eppendorf tube, taking care to avoid contamination with urine or other substances. Two aliquots were collected from each participant, and these were stored at −80°C.

#### DNA Extraction and Quality Testing

Stool samples were thawed, and fecal microbial genomic DNA was extracted using a MagPure Stool DNA KF Kit B, according to the manufacturer’s instructions. A Qubit^®^ dsDNA BR Assay Kit was used to accurately quantify the DNA concentrations. One-percent agarose gel electrophoresis (150 V for ∼40 min) was used to check the quality of the DNA.

#### 16s rDNAV3–V4 Segment Amplification

The primer sequences used for the polymerase chain reaction (PCR) reaction were 341F (5′-ACTCCTACGGGAGGCAGCAG-3′) and 806R (5′-GGACTACHVGGGTWTCTAAT-3′). The 16S libraries were sequenced at the Analytical Genomics Core of Sanford Burnham Prebys Medical Discovery Institute (Lake Nona, FL, United States) and the Beijing Genomics Institute (Beijing, China). The original FASTQ files were processed using the novel 16S amplicon sequencing pipeline HiMap^1^ (bioRxiv 565572), which generates Operational Strain Unit as its output. The read counts were then converted to relative abundances. Log10-transformed relative abundances were used for comparisons of samples from each group.

#### PCR Product Purification

The PCR products were purified using Agencourt AMPure XP magnetic beads, dissolved in Elution Buffer, labeled, and used for library construction.

#### Library Quality Inspection

An Agilent 2100 Bioanalyzer was used to characterize the sizes and concentrations of the fragments that constituted the libraries. Qualifying libraries were sequenced on the Illumina HiSeq platform according to the size of the inserted fragments and using the Illumina standard pipeline, generating read areas of 2 × 300 bp.

#### Bioinformatics Analysis

##### Data preprocessing

Off-machine data filtering was performed to remove low-quality, joint pollution, N, and low-complexity reads. The filtered reads were spliced using Fast Length Adjustment of Short reads (FLASH v1.2.11). The minimum matching length was 15 bp, and the allowable mismatch rate in the overlapping area was 0.1. The reads were then spliced into tags using the overlaps between the reads.

##### Operational taxonomic unit cluster analysis

To facilitate analysis, certain taxonomic units were identified using unified marks, which are used in the study of phylogeny and population genetics, and were referred to as operational taxonomic units (OTUs). To study all the bacterial taxa that were sequenced in a sample, the sequences were classified into groups according to their similarity, and each group represented an OTU. In general, tags with a similarity of > 97% were clustered into an OTU.

We used Venn diagrams to demonstrate the numbers of common and unique OTUs for a variety of samples and also visually displayed the OTU overlap between the samples graphically. Partial least-squares discrimination analysis (PLS-DA) is a method of multivariate statistical analysis and a method of supervision that is used for discriminant analysis, which reflects the differences between groups to the greatest extent. Various colors and shapes were used to represent the sample groups under various conditions.

##### Analysis of species composition

We used the RDP classifier Bayesian algorithm to perform taxonomic analysis of representative sequences of each OTU to obtain species classification information corresponding to each OTU. The community compositions of the SAA and healthy control groups were analyzed at the levels of phylum, class, order, family, genus, and species and are displayed in the form of abundance histograms. R software (v3.4.1) was used to analyze the relative abundance graphs for the two groups.

##### Alpha diversity analysis

Alpha diversity is a means of assessing the species diversity of an individual sample and is described in the form of the Chao index, observed species index, Ace index, Simpson index, Shannon index, and good-coverage index. The Chao, Ace, and observed species indices were used to describe the species richness of the samples, and the Simpson and Shannon indices were used to describe the species diversity of the samples, which comprises the species richness and species evenness. When the species richness is the same, the species evenness in the community is proportional to the species diversity. Therefore, the larger the Chao, Ace, observed species, and Shannon indices, and the smaller the Simpson index, the higher the species diversity of the sample. The good-coverage index reflects the coverage of the sample library, and its value is inversely proportional to the probability that the sequence was measured in the sample, such that the higher its value is, the more representative it is of the real composition of the sample.

##### Differential species analysis

The Wilcoxon rank-sum and Kruskal–Wallis tests were used to identify significant differences in the abundance of microbial species between the groups (*p* < 0.05).

##### Association analysis and model prediction

Species that were present at differing abundances according to the results of rank-sum tests were analyzed using a Spearman correlation heatmap of the dominant species, drawn using R software. Important patterns and relationships between the dominant species are identified by color: the darker the color is, the stronger the correlation is between the species.

### Plasma Metabolomic Analysis

Fresh whole-blood samples (5 mL) were collected from the patient and control groups, placed into EDTA anticoagulation tubes, and centrifuged at 1,600 × *g* for 10 min at 4°C to separate the plasma. All the samples were analyzed using ultrahigh-performance liquid chromatography (UPLC) (Waters, United Kingdom), an Acquity UPLC BEH C18 column (100 mm × 2.1 mm, 1.7 μm, Waters, United Kingdom) for reverse-phase separation, and a high-resolution tandem mass spectrometer [Xevo G2 XS quadrupole-time of flight (Q-TOF), Waters, United Kingdom] to identify metabolites eluted from the column. The Q-TOF was operated in both positive and negative ion modes.

### Statistical Analysis

Student’s *t*-test, fold-change analysis, and PLS-DA were used to identify metabolites that were present at differing concentrations in the SAA and control groups. Differences were considered statistically significant when there was a fold difference ≥ 1.2 or ≤ 0.8333 and a *p* < 0.05.

## Results

### Composition of the Intestinal Microbiota of Patients With SAA and Controls

#### OTU Analysis

**FIGURE 1:**
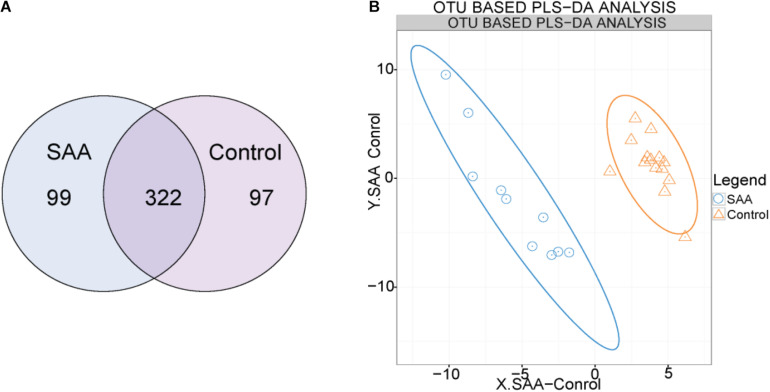
**(A)** Venn diagram of the OTUs (A: SAA group, N: control group). **(B)** Out-based PLS-DA analysis. Orange triangles represent intestinal microbial samples from the control group, and blue circles represent samples from patients with SAA.

OTU clustering was performed with a sequence similarity of 97%. The Venn diagram of the OTUs shows that the healthy control group contained a total of 419 OTUs, the SAA group contained 421 OTUs, and the two groups shared 322 OTUs (Figure 1A). Furthermore, we performed PLS-DA analysis in the R (v3.2.1) mixOmics package to analyze the OTU data, which showed that patients with SAA had a different intestinal microbiome to the healthy controls (Figure 1B).

#### Alpha Diversity Analysis

**FIGURE 2:**
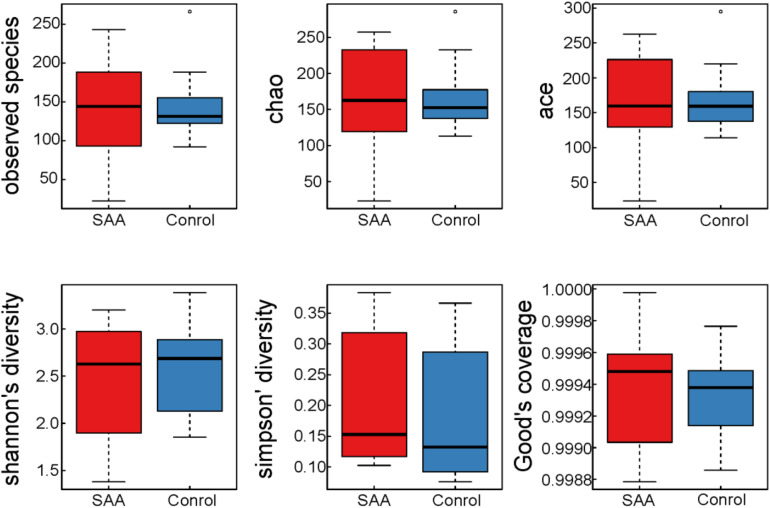
Boxplots of indices of the alpha diversity of the intestinal microbiota in the SAA and control groups.

The mean good coverage values for the SAA and healthy control groups were > 99%, indicating that the sequences obtained for each sample covered almost all the bacterial sequences in the library. As shown in Figure 2, the values of the Chao, Observed species, Ace, and Shannon indices for the SAA group were slightly lower than those for the healthy control group, and the Simpson index was higher (Figure 2 and Table 2).

**TABLE 2:** Results of the richness and diversity analysis of the intestinal microbiota.

Alpha diversity index	SAA (*n* = 10)	Normal control (*n* = 14)	*p*-value
Sobs	135.1	143.21429	0.52977
Chao	161.60401	163.85759	0.80945
Ace	164.78136	168.27365	0.83967
Shannon	2.39946	2.57708	0.18377
Simpson	0.20437	0.18683	0.23579
Coverage	0.99937	0.99934	0.95995

### Structures of the Microbiota of Patients With SAA and Controls

#### Intestinal Microbial Composition of the SAA and Control Groups at the Phylum Level

**FIGURE 3:**
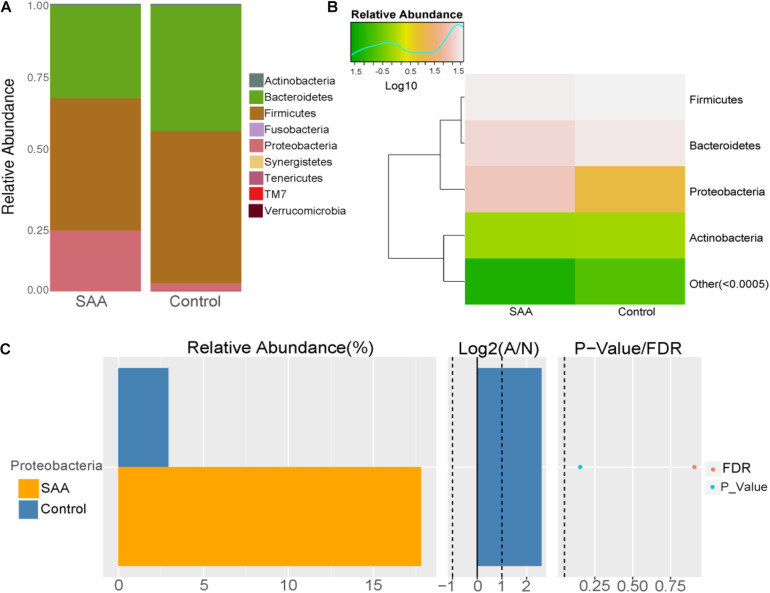
Intestinal microbial compositions of the SAA and control groups at the phylum level. **(A)** Bar plot, **(B)** heatmap, **(C)** Wilcoxon rank-sum test.

Composition analysis showed that the relative abundance of *Proteobacteria* in the intestinal microbiota was higher in the SAA group than in the control group, but that the relative abundance of *Bacteroidetes* was lower (Figures 3A,B). However, there were no significant differences in differential microbial phylum between the SAA group and control groups, according to the Wilcoxon rank-sum *t*-test (Figure 3C).

#### Intestinal Microbial Composition of the SAA and Control Groups at the Class Level

**FIGURE 4:**
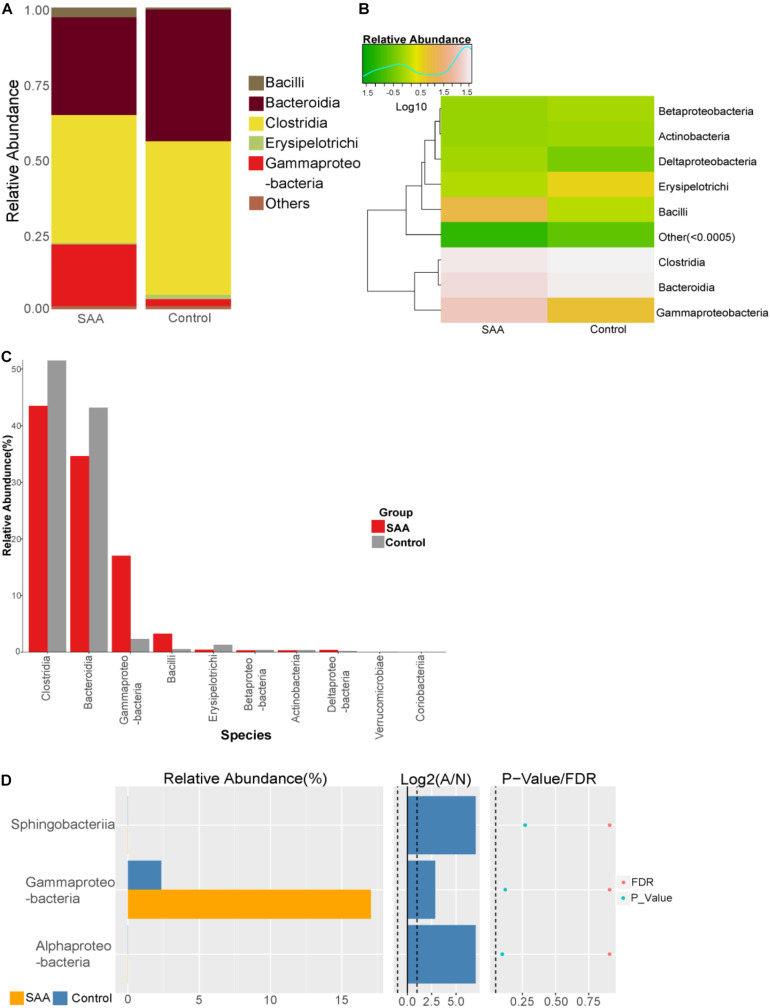
Intestinal microbial compositions of the SAA and control groups at the class level. **(A)** Bar plot, **(B)** heatmap, **(C)** top 10 most abundant species, **(D)** Wilcoxon rank-sum test results.

Composition analysis showed that the relative abundances of *Bacillus* and *Gammaproteobacteria* in the intestinal microbiota were higher in the SAA group than in the control group, but that the relative abundance of *Bacteroidia* was lower (Figures 4A,B). Further analysis of the top 10 most abundant species in the SAA and control groups showed that there were no significant differences between the groups (Kruskal–Wallis and Wilcoxon rank-sum tests) (Figures 4C,D).

#### Intestinal Microbial Composition of the SAA and Control Groups at the Order Level

**FIGURE 5:**
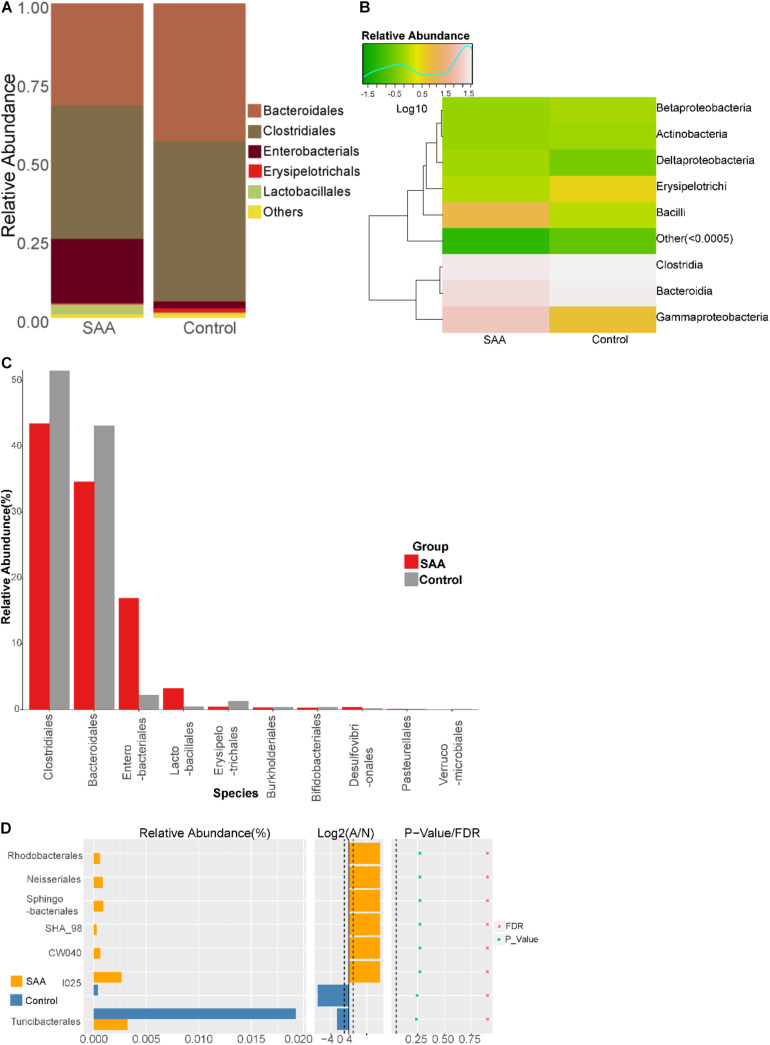
Intestinal microbial compositions of the SAA and control groups at the order level. **(A)** Bar plot, **(B)** heatmap, **(C)** top 10 most abundant species, **(D)** Wilcoxon rank-sum test results.

Composition analysis showed that the relative abundances of *Enterobacteriales* and *Lactobacillales* in the intestinal microbiota were higher in the SAA group than in the control group, but the relative abundances of *Bacteroidales*, *Clostridiales*, and *Erysipelotrichales* were lower (Figures 5A,B). Further analysis of the top 10 most abundant species in the SAA and control groups showed that the abundance of *Enterobacteriales* was higher in the SAA group than in the control group (*p* < 0.05), according to the Kruskal–Wallis test (Figure 5C). However, there were no other significant differences between the two groups (Wilcoxon rank-sum test) (Figure 5D).

#### Intestinal Microbial Composition of the SAA and Control Groups at the Family Level

**FIGURE 6:**
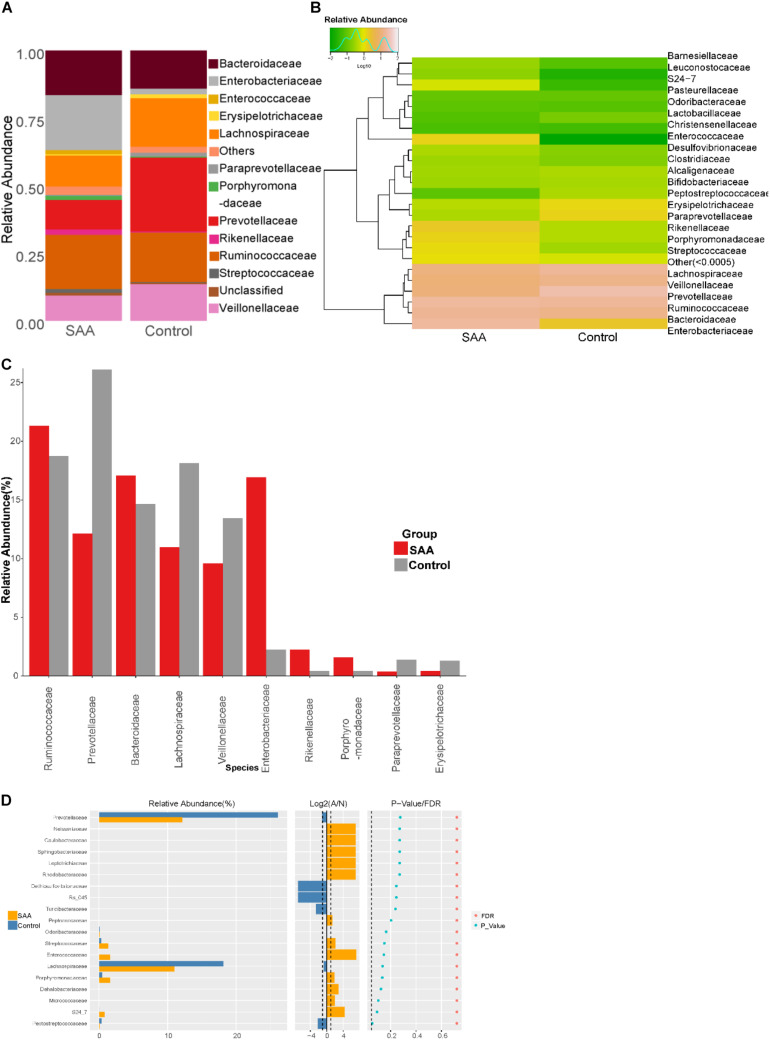
Intestinal microbial compositions of the SAA and control groups at the family level. **(A)** Bar plot, **(B)** heatmap, **(C)** top 10 most abundant species, **(D)** Wilcoxon rank-sum test results.

Composition analysis showed that the relative abundances of *Ruminococcaceae* and *Paraprevotellaceae* in the intestinal microbiota were higher in the SAA group than in the control group (Figures 6A,B). Further analysis of the top most abundant 10 species in the two groups showed that the abundance of the *Enterobacteriaceae* was higher in the SAA group than in the control group (*p* < 0.05), according to the Kruskal–Wallis test (Figure 6C). However, there were no other significant differences between the two groups (Wilcoxon rank-sum test) (Figure 6D).

#### Microbial Composition of the SAA and Control Groups at the Genus Level

**FIGURE 7:**
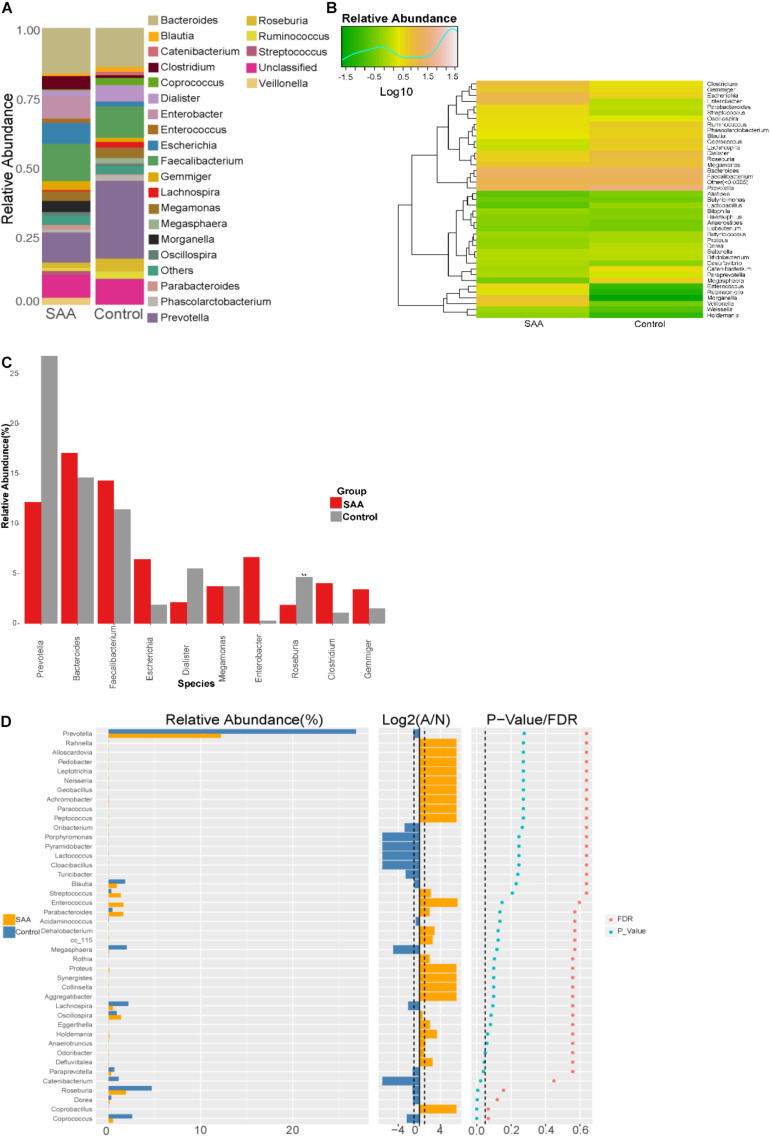
Intestinal microbial compositions of the SAA and control groups at the genus level. **(A)** Bar plot, **(B)** heatmap, **(C)** top 10 most abundant species, **(D)** Wilcoxon rank-sum test results.

Composition analysis showed that the relative abundances of *Clostridium*, *Escherichia, Morganella*, and *Veillonella* in the intestinal microbiota were higher in the SAA group than in the control group, but the relative abundances of *Coprococcus* and *Roseburia* were lower (Figures 7A,B). Further analysis of the top 10 most abundant species in the two groups showed that the abundance of *Roseburia* was lower in the SAA group (*p* < 0.05; Wilcoxon rank-sum test) (Figures 7C,D).

#### Intestinal Microbial Composition of the SAA and Control Groups at the Species Level

**FIGURE 8:**
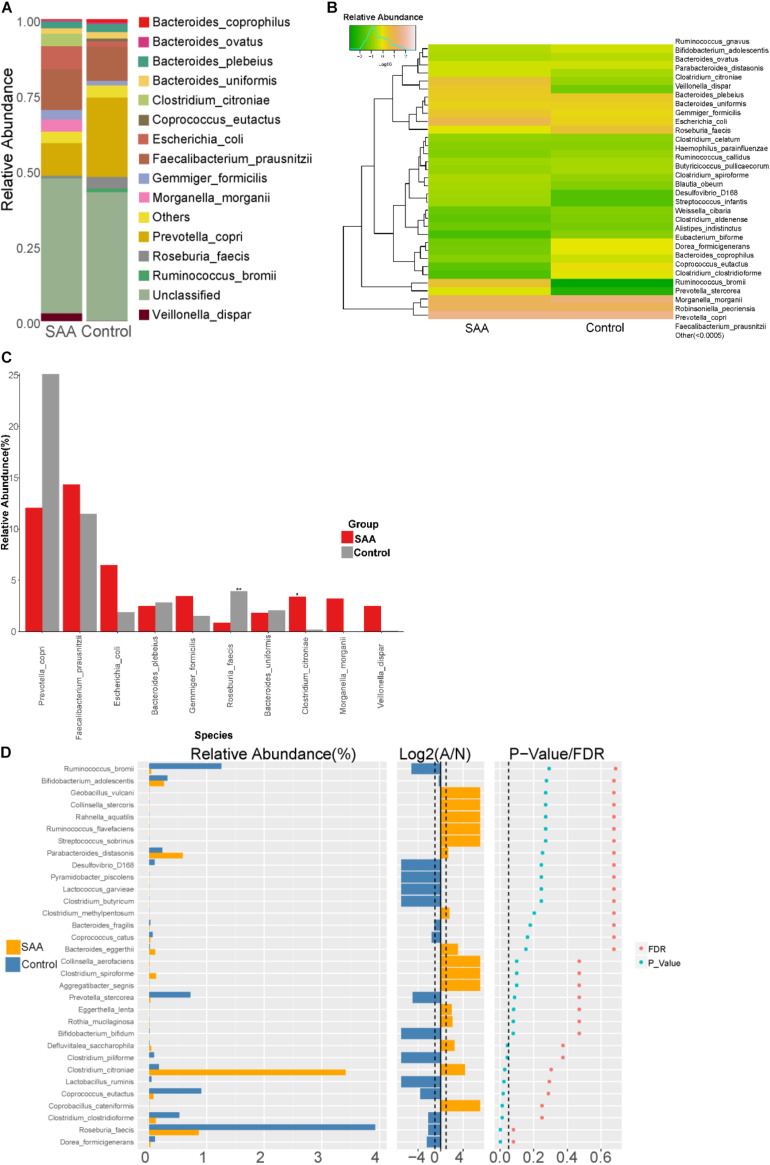
Intestinal microbial compositions of the SAA and control groups at the species level. **(A)** Bar plot, **(B)** heatmap, **(C)** Wilcoxon rank-sum test results, **(D)** top 10 most abundant species.

Composition analysis showed that the relative abundances of *Gemmiger formicilis*, *Escherichia coli*, *Clostridium citroniae*, *Morganella morganii*, and *Veillonella dispar* in the intestinal microbiota were higher in the SAA group than in the control group, but the relative abundances of *Bacteroides coprophilus*, *Coprococcus eutactus*, *Prevotella copri*, *Roseburia faecis*, and *Ruminococcus bromii* were lower (Figures 8A,B). Further analysis of the two groups showed that the abundances of *C. citroniae* and *Coprococcus cateriicrmis* were higher, and those of *R. faecis*, *C. eutactus*, *Clostridium clostridioforme*, *Lactobacillus ruminis*, and *P. copri* were lower in the SAA group (all *p* < 0.05; Wilcoxon rank-sum test) (Figure 8C). In addition, the SAA group showed higher abundance of *C. citroniae* and lower abundance of *R. faecis* among the top 10 most abundant species (Kruskal–Wallis test) (Figure 8D).

#### Correlation Analysis and Model Prediction

**FIGURE 9:**
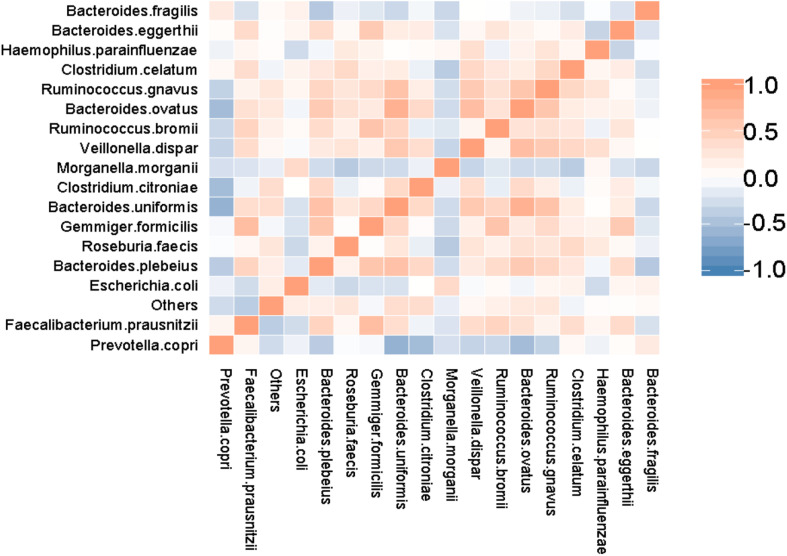
Heatmap showing the results of the association analysis for the SAA and control groups.

The correlations between the associated dominant species in the SAA and control groups are shown in the form of a heatmap (Figure 9). This shows a strong competitive relationship between *P. copri* and *C. citroniae*.

### Findings of the Metabolomic Analysis

**FIGURE 10:**
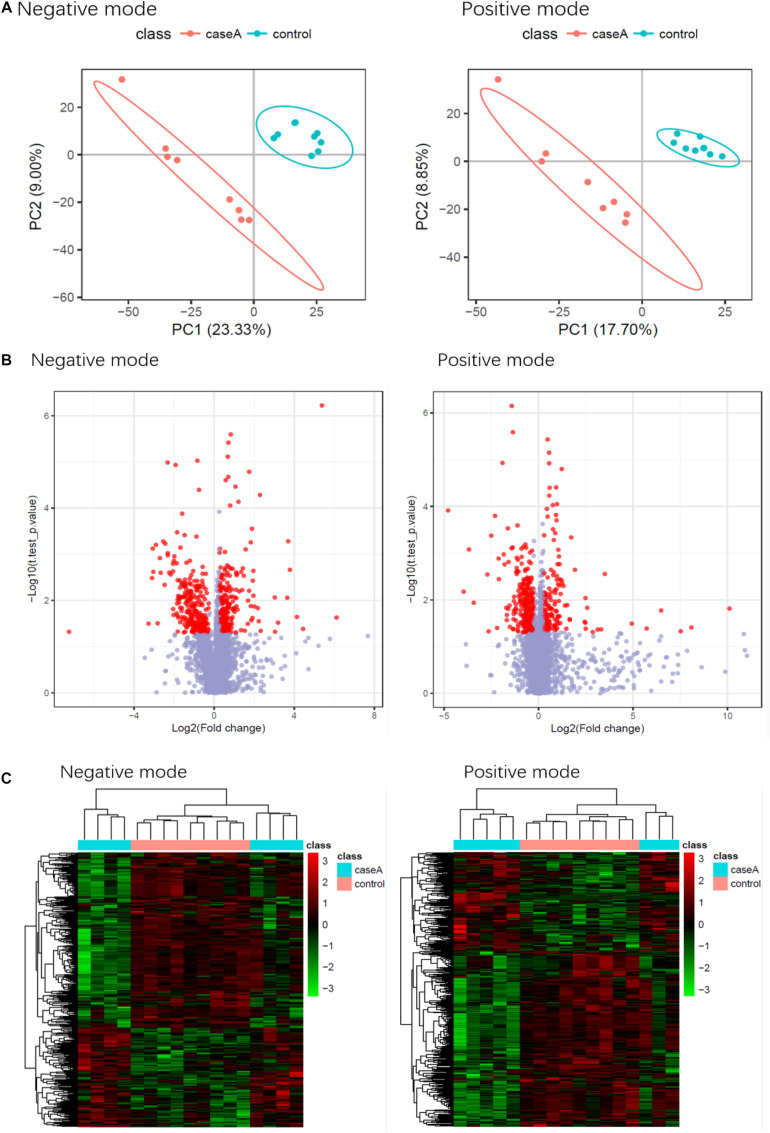
Results of the metabolomic comparison of patients with SAA and healthy controls in both positive and negative mass spectrometric ion modes. **(A)** PLS-DA, **(B)** Visual volcanic map, **(C)** heatmap.

The metabolomic analysis showed that patients with SAA had a different metabolite profile to that of healthy controls. The results are displayed using PLS-DA (Figure 10A), a visual volcanic map (Figure 10B), and a heatmap (Figure 10C). Differences were present in both the positive and negative mass spectrometric ion modes.

Eight metabolites were found to be present at significantly different plasma concentrations in the SAA and healthy control groups by the screening and identification of derived ions. Coumaric acid, L-phenylalanine, and sulfate were present at higher concentrations in the SAA group, whereas L-glutamic γ-semialdehyde, theobromine,3a, 7a-dihydroxy-5b-cholestane, γ-δ-dioxovaleric acid, and (12Z)-9, 10-dihydroxyoctadec-12-enoic acid were present at lower concentrations (Table 3). These metabolites are amino acids, steroids, keto acids, fatty acids, or hydroxyphenylacrylic acid, or derivatives thereof.

**TABLE 3:** List of metabolites that were present at differing plasma concentrations in the SAA and healthy control groups.

Description	Molecular formula	m/z	Ion mode	Matching degree	Fold change
Coumaric acid	C_9_H_8_O_3_	182.08	ESI^+^	93.6	1.26
L-Phenylalanine	C_9_H_11_NO_2_	188.07	ESI^+^	59.7	1.68
Sulfate	H_2_O_4_S	96.96	ESI^–^	86.9	1.36
L-Glutamic γ-semialdehyde	C_5_H_9_NO_3_	132.07	ESI^+^	68.2	0.80
Theobromine	C_7_H_8_N_4_O_2_	181.07	ESI^+^	54.9	0.08
3a, 7a-Dihydroxy-5b-cholestane	C_27_H_48_O_2_	405.37	ESI^+^	54.7	0.52
γ-δ-Dioxovaleric acid	C_5_H_6_O_4_	129.02	ESI^–^	82.2	0.59
9, 10-DHOME	C_18_H_34_O_4_	313.24	ESI^–^	53.6	0.57

## Discussion

SAAs are a class of highly heterogeneous hematological diseases that have complex etiologies and pathogenesis. The clinical symptoms often include fatal anemia, hemorrhage, and infection. SAA is considered to be an immune disorder, and in previous studies, the number of activated CD8^+^ T cells has been shown to be higher in patients with SAA (Sheng et al., 2014; Xiao et al., 2017). Hyperfunctional T cells have a significant inhibitory effect on bone marrow hematopoiesis *in vitro* (Young et al., 2010). Gidvani et al. (2007) aimed to identify single-nucleotide polymorphisms (SNPs) in genes encoding cytokines associated with autoimmune diseases, such as interleukin 6 (IL-6), IL-10, tumor necrosis factor α (TNF-α), interferon γ (IFN-γ), and transforming growth factor β1, and found that SNPs, especially of the genes encoding TNF-α and IFN-γ, were present in patients with SAA, which suggests that these genes might be involved in the pathogenesis of SAA. Solomou et al. (2006) measured the number of Th1 and Th2 cells in the peripheral blood of patients with SAA before and after IST and found that the Th1/Th2 ratio was abnormal. The abnormally large number of Th1 cells is considered to play an important role in the immunopathogenesis of SAA (Du et al., 2013). Solomou et al. (2007) studied the Tregs of patients with SAA and found that the expression of FoxP3 is lower in CD4^+^CD25^+^ cells, which suggests that patients with SAA have poor immune tolerance. In our previous studies, we showed that the mDCs of patients with SAA are hyperfunctional (Zonghong et al., 2011), that there were fewer natural killer cells in such patients, and that the number of natural killer cells recovered after IST (Liu et al., 2014). Therefore, SAA is an autoimmune disease characterized by hyperfunctional T lymphocyte–mediated bone marrow damage, which is associated with poor hematopoiesis and immune tolerance. However, the etiology of the defects in the immune system is unclear.

It has recently been shown that metabolism and the intestinal microbiota play important roles in autoimmune diseases. Small-molecule metabolites generated by gut bacteria have biological activities and affect host health (Nicholson et al., 2012). Metabolomic studies of the differences in metabolite concentrations that are associated with internal and external factors have aided understanding of the pathophysiology of diseases including systemic lupus erythematosus (Yan et al., 2016), rheumatoid arthritis (RA) (Narasimhan et al., 2018), and systemic sclerosis (Bengtsson et al., 2016).

In the present study, patients with SAA and healthy controls had differing intestinal microbial compositions. Specifically, *Enterobacteriales* was more abundant in the intestine of patients with SAA. Some previous studies have shown that larger numbers of *Enterobacteriales* are associated with poor intestinal barrier function, which permits antigens from the diet or bacteria to enter the circulation from the intestine and activate the immune system (Pedersen et al., 2018). It has been speculated that *Enterobacteriales* may be involved in the immune and metabolic defects involved in SAA. We also found larger numbers of *C. citroniae* and *C. cateriicrmis* and smaller numbers of *R. faecis*, *C. eutactus*, *C. clostridioforme*, *L. ruminis*, and *Dorea formicigenerans* in the intestines of patients with SAA. The differences in the abundances of *C. citroniae* and *R. faecis* were particularly marked. *Roseburia* species, including *R. faecis*, can produce short-chain fatty acids (SCFAs), and especially butyrate. It has previously been shown that SCFAs have anti-inflammatory effects through regulation of immune cell chemotaxis and reactive oxygen species release, which inhibits the production of the proinflammatory molecules TNF-α, IL-1β, and nitric oxide, and the activity of nuclear factor κB. In addition, butyrate inhibits the production of IL-2 and lymphocyte proliferation (Säemann et al., 2000; Cavaglieri et al., 2003; Ni et al., 2010), maintains intestinal health and immune defense, participates in the regulation of Tregs, and plays an important role in the maturation of the immune system (Jost et al., 2013). It can also reduce injury to the colon and the symptoms of inflammatory bowel disease (Tamanai-Shacoori et al., 2017). Furthermore, butyrate generated by gut microbes can also enter the circulation and improve myeloid hematopoiesis. There is also evidence that *Roseburia* can produce bacteriocin-like substance, which is a polypeptide that has antibacterial activity and helps prevent infection with a variety of pathogens (Hatziioanou et al., 2013). Thus, a lower abundance of *R. faecis* might lead to greater production of proinflammatory factors in SAA. *C. citroniae* is a pathogen that is associated with bacteremia and celiac infection. Therefore, the greater abundance of *C. citroniae* may imply that patients with SAA are at a higher risk of infections that would activate the immune response. Taken together, these findings imply that the abnormal immune response that characterizes SAA might be ameliorated by measures that affect the composition of the intestinal microbiota.

The results of species association analysis in the two groups showed that there was strong competition between *P. copri* and *C. citroniae*, which may have implications for the immune system. *P. copri* is a member of the *Bacteroidetes*, which can produce succinate, a metabolic regulator and participant in proinflammatory responses. Previous studies have shown that the relative abundance of *P. copri* in the intestinal tract of patients with RA is high, but that in early RA it is lower than that in healthy individuals (Maeda et al., 2016). Therefore, the importance of *P. copri* in patients with RA requires further study. In addition, it has been shown that the relative abundance of *P. copri* is lower in the intestines of patients with psoriasis, which may be related to their abnormal immunity (Tamanai-Shacoori et al., 2017). In the present study, the relative abundance of *P. copri* was lower in the SAA group than in the healthy control group, which may have influenced the immune system of the SAA group. The lower abundance of *P. copri* and the higher abundance of *C. citroniae* broke the equilibrium competition relationship, which may also have had an influence on the immunity of patients with SAA.

Alpha diversity analysis showed that the composition of the microbiota of patients with SAA differed from that of normal controls, but probably owing to the small sample size, a significant difference was not identified. Therefore, future studies should be conducted that recruit larger numbers of patients and controls.

Previous metabolomic studies have generated insights into hematological diseases (Chen et al., 2014). In this previous study, a prognosis risk score was created using six metabolite markers that are indicative of upregulation of glycolysis and the tricarboxylic acid cycle, and an upregulation of glycolysis contributes to a lower sensitivity to cytarabine. In the present study, we found that L-phenylalanine, coumaric acid, and sulfate were present in higher concentrations in the plasma of patients with SAA, which suggests that these metabolites may be useful for the characterization of SAA. Phenylalanine is an essential aromatic amino acid that is necessary for the synthesis of neurotransmitters and hormones. However, high concentrations of phenylalanine are neurotoxic and increase the risk of cardiovascular disease (Wurtz et al., 2015), although the mechanisms involved have yet to be characterized. In the present study, the plasma phenylalanine concentration was high in patients with SAA, and this might have been responsible for bone marrow hematopoietic stem cell damage.

## Conclusion

In conclusion, the plasma concentrations of certain metabolites and the composition of the intestinal microbiota are altered in patients with SAA. The abnormalities in the metabolites may be associated with the intestinal dysbacteriosis and might indicate potential molecular mechanisms for the immune defects that characterize SAA. Furthermore, these substances might represent candidate metabolic markers of SAA and/or suggest novel therapeutic targets.

## Data Availability Statement

The microbiome sequence data have been deposited in the National Center for Biotechnology Information (NCBI) Sequence Read Archive (SRA) under BioProject PRJNA524870. The gene expression data generated by the NanoString analysis has been deposited in the GEO database under the accession number GSE127753.

## Ethics Statement

The studies involving human participants were reviewed and approved by Medical Ethics Committee of General Hospital of Tianjin Medical University. Written informed consent to participate in this study was provided by the participants’ legal guardian/next of kin.

## Author Contributions

All authors listed have made a substantial, direct and intellectual contribution to the work, and approved it for publication.

## Conflict of Interest

The authors declare that the research was conducted in the absence of any commercial or financial relationships that could be construed as a potential conflict of interest.

## Publisher’s Note

All claims expressed in this article are solely those of the authors and do not necessarily represent those of their affiliated organizations, or those of the publisher, the editors and the reviewers. Any product that may be evaluated in this article, or claim that may be made by its manufacturer, is not guaranteed or endorsed by the publisher.

## Footnotes

1
http://github.com/taolonglab/himap
